# Combination of Quercetin and 2-Methoxyestradiol Enhances Inhibition of Human Prostate Cancer LNCaP and PC-3 Cells Xenograft Tumor Growth

**DOI:** 10.1371/journal.pone.0128277

**Published:** 2015-05-26

**Authors:** Feiya Yang, Liming Song, Huiping Wang, Jun Wang, Zhiqing Xu, Nianzeng Xing

**Affiliations:** 1 Department of Urology, Beijing Chaoyang Hospital, Capital Medical University, Beijing, P.R. China; 2 Department of Reproductive Immunology and Pharmacology, National Research Institute for Family Planning, Beijing, P.R. China; 3 Department of Neurosurgery, the First hospital of China Medical University, Shenyang, P.R. China; 4 Institute of Neuroscience, Beijing Key Laboratory of Neural Regeneration and Repair, Capital Medical University, Beijing, P.R. China; II Università di Napoli, ITALY

## Abstract

Quercetin and 2-Methoxyestradiol (2-ME) are promising anti-cancer substances. Our previous *in vitro* study showed that quercetin synergized with 2-Methoxyestradiol exhibiting increased antiproliferative and proapoptotic activity in both androgen-dependent LNCaP and androgen-independent PC-3 human prostate cancer cell lines. In the present study, we determined whether their combination could inhibit LNCaP and PC-3 xenograft tumor growth *in vivo* and explored the underlying mechanism. Human prostate cancer LNCaP and PC-3 cells were inoculated subcutaneously in male BALB/c nude mice. When xenograft tumors reached about 100 mm^3^, mice were randomly allocated to vehicle control, quercetin or 2-Methoxyestradiol singly treated and combination treatment groups. After therapeutic intervention for 4 weeks, combination treatment of quercetin and 2-ME i) significantly inhibited prostate cancer xenograft tumor growth by 46.8% for LNCaP and 51.3% for PC-3 as compared to vehicle control group, more effective than quercetin (28.4% for LNCaP, 24.8% for PC3) or 2-ME (32.1% for LNCaP, 28.9% for PC3) alone; ii) was well tolerated by BALB/c mice and no obvious toxic reactions were observed; iii) led to higher Bax/Bcl-2 ratio, cleaved caspase-3 protein expression and apoptosis rate; and iv) resulted in lower phosphorylated AKT (pAKT) protein level, vascular endothelial growth factor protein and mRNA expression, microvascular density and proliferation rate than single drug treatment. These effects were more remarkable compared to vehicle group. Therefore, combination of quercetin and 2-ME can serve as a novel clinical treatment regimen owning the potential of enhancing antitumor effect on prostate cancer *in vivo* and lessening the dose and side effects of either quercetin or 2-ME alone. These *in vivo* results will lay a further solid basis for subsequent researches on this novel therapeutic regimen in human prostate cancer.

## Introduction

Prostate cancer is the second leading cause of cancer mortality in males, with an estimated 233,000 new cases and 29,480 deaths in United States in 2014 [1]. Although it can be cured by radical prostatectomy or radiation at early stage, most patients will suffer from local recurrence and distant metastasis later [2]. After effective treatment of androgen ablation in the first 1–3 years, it generally develops into castration-resistant prostate cancer (CRPC) characterized with elevated prostate specific antigen (PSA), higher metastasis rate, more aggressiveness, etc [3]. Combination of docetaxel and prednisone, the standard first-line systemic chemotherapy for CRPC, is not curative and only prolongs overall survival time for a short period. Moreover, it makes patients suffer from severe side effects [4]. Therefore, it is of urgent need to develop novel drug therapies that can overcome these shortcomings working singly or in combination for CRPC.

Quercetin (3, 3’, 4’, 5, 7-pentahydroxyflavone, Que), a bioactive flavonoid abundant in vegetables and fruits especially in onions, apples, tea and red wine, has exhibited promising anti-tumor property in many human cancer cells including prostate cancer both in vitro and in vivo [5,6]. However, due to low bioavailability, its anti-tumor effect has been restricted to a great extent. In order to overcome this deficiency, quercetin has been combined with other anti-cancer drugs to enhance inhibition of various tumors including prostate cancer [6,7,8].

2-Methoxyestradiol (2-ME), a natural endogenous derivative of 17β-estradiol (E2) rarely exhibiting estrogenic activity, has been reported to demonstrate anti-cancer action in a wide range of tumors [9]. As to prostate cancer, 2-ME can inhibit both androgen-dependent and androgen-independent cancer cells in vitro and in vivo [10,11]. Nevertheless, 2-ME has the disadvantage of limited biological accessibility and rapid degradation [12]. So as to solve the problem, drug combination has been proposed and aroused increased anti-cancer effect with less side effects compared to single drug treatment [10,13].

Therefore, despite the common disadvantage of low bioavailability for both quercetin and 2-ME, it seems optimistic when they were combined with other substances owing cancer treated effect. Previously, we conducted a combined use of quercetin and 2-ME in both androgen-dependent LNCaP and androgen-independent PC-3 human prostate cancer cell lines and found a synergistic antiproliferative and proapoptotic effect compared with quercetin or 2-ME alone [14]. The present study was to investigate the combined effect of quercetin and 2-ME on LNCaP and PC-3 xenograft tumor in vivo and study the mechanism for the first time.

## Materials and Methods

### Chemical reagents

Quercetin, 2-Methoxyestradiol, hydroxypropyl-β-cyclodextrin (HPβCD) and carboxymethyl cellulose (CMC) were purchased from Sigma (St. Louis, MO, USA). RPMI-1640 medium, 0.25% trypsin-EDTA and fetal bovine serum were got from Hyclone (Logan, UT, USA), and Matrigel was from BD Biosciences (Franklin Lakes, NJ, USA).

### Cell Lines and Cell Culture

Human prostate cancer androgen-dependent LNCaP and androgen-independent PC-3 cell lines (two commonly used cell lines that has been used by many other researchers [15,16,17]), obtained from Peking Union Medical College, were cultured in RPMI-1640 medium (Hyclone, Logan, UT) supplemented with 10% fetal bovine serum (Hyclone) and placed in incubator containing 95% air and 5% CO_2_ at 37°C.

### Animal study

All experiment procedures related to animals were approved by the Committee of Animal Experimentation and the Ethic Committee of Capital Medical University (Permit Number: 2013-X-83). Male BALB/c nude mice 4–6 weeks old were provided by the ministry of experimental animals of Capital Medical University and kept in pathogen free environment with 12-hour light/dark cycle, controlled humidity and temperature. Mice were allowed to get accustomed to new environment for one week before commencement of experiment.

Before the formal in vivo experiment, we evaluated the toxicity of two combined drugs and vehicle that would be administrated simultaneously using two groups of male BALB/c nude mice (n = 5 each). Solvent for quercetin was 25% hydroxypropyl-β-cyclodextrin (HPβCD, w/v in ddH_2_O) and for 2-Methoxyestradiol was 25% HPβCD containing 0.5% carboxymethyl cellulose (CMC, w/v in ddH_2_O) [10,18]. Drug group were given the two drugs, namely dissolved quercetin and 2-ME, and vehicle control group were given two drug-free vehicles, namely 25% HPβCD containing or not containing 0.5% CMC. After operation, toxic reaction was observed in the mice of both groups represented as poor mental state, lightly twisting the body, convulsion and occasional moderate haematuria that were in consistent with the description of Ehteda A and may be attributed to high concentration of HPβCD [10]. For this reason, in the subsequent experiment, combination of quercetin and 2-ME was carried out in this way: quercetin was given on day 1, followed by 2-ME given on day 2.

Mice were inoculated subcutaneously with 5×10^5^ PC-3 cells suspended in 100μL PBS and 2×10^8^ LNCaP cells suspended in 100μL of matrigel and PBS mixture (1:1) on the right back. When xenograft tumors reached a volume of approximately 100mm^3^, mice were randomly assigned to four groups (n = 8 each group) and treated intraperitoneally. Therapeutic schedule based on our in vitro results, preliminary experiments and many other researchers' studies was as follows: (1) Vehicle control group: vehicle of quercetin on day 1, vehicle of 2-ME on day 2, (2) Quercetin treated group: quercetin 75mg/kg on day 1, vehicle of 2-ME on day 2, (3) 2-ME treated group: vehicle of quercetin on day 1, 2-ME 150mg/kg on day 2, (4) Combination treatment group: quercetin 75mg/kg on day 1, 2-ME 150mg/kg on day 2. Two days was a treatment cycle and the whole treatment process lasted for 4 weeks [13,19,20,21,22,23]. Tumor sizes were monitored every 2 days using caliper and tumor volume were calculated according to the formula: L×S^2^×0.5, in which L represents the longest diameter and S represents the shortest diameter of tumor [10]. Mice were weighed as well. At the end of treatment procedure, on day 29, mice were anesthetized with chloral hydrate and sacrificed by cervical dislocation. Xenograft tumors were taken out quickly and weighed. One part of it was put into liquid nitrogen immediately for future biomarker analysis and the other part was fixed in 10% neutral buffered formalin for immunohistochemical analysis. Serum biochemical parameters such as ALT, AST, creatinine and urea nitrogen that reflected drug toxicity were also detected.

### Western blot analysis

Tumor tissues were mixed with RIPA Lysis buffer (Applygen Inc., Beijing, China) containing protease inhibitor cocktail (Roche, Switzerland). Lysates were centrifuged and supernatant was collected. After quantified using BCA protein assay kit (Pierce, Rockford, USA), 80 μg protein was separated by 6%-12% SDS-PAGE and transferred to polyvinylidene fluoride (PVDF) membrane (Pall, NY, USA) which was then blocked by 5% non-fat milk and incubated with primary antibodies: Bcl-2 (1:1000, Cell Signaling, Beverly, MA), Bax (1:1000, Cell Signaling), Caspase-3 (1:1000, Cell Signaling), AKT and pAKT (1:1000, Cell Signaling), VEGF (1:500, Abcam) at 4°C overnight, GAPDH (1:10000, sigma) at room temperature for 1hour, followed by horseradish peroxidase conjugated secondary antibodies (1:2000, Cell Signaling) incubation for another 1 hour at room temperature. The antigen-antibody complex bands were detected by enhanced chemiluminescence kit (ECL-Plus, Amersham Pharmacia Biotech, Piscataway, NJ, USA). GAPDH was used as loading control.

### Reverse transcription-quantitative real time polymerase chain reaction (RT-qPCR)

Total RNA of tumor tissue was extracted using Trizol reagent (Invitrogen, USA) and first-strand cDNA was synthesized using Transcriptor First Strand cDNA Synthesis Kit (Roche, Germany) with oligo-(dT) primers. Sequences of VEGF and β-actin primers were as follows: Human VEGF: Forward: 5'-TCTACCTCCACCATGCCAAGT-3', reverse: 5'-GATGATTCTGCCC TCCTCCTT-3'. β-actin: Forward: 5'-CGGGAAATCGTGCGTGAC-3', reverse: 5'- GTGGCCAT CTCTTGCTCGAA-3'. Accuracy of PCR products was verified by DNA sequencing.

Total 20 μL reaction mixture consisted of 10 μL SYBR green PCR master mix (Applied Biosystems), 8 μL ddH_2_O, 1μL cDNA template and 1μL primers reacted following the thermal cycling conditions: one cycle at 50°C for 2 minutes, followed by 95°C for 10 minutes, 40 cycles of 95°C for 20 seconds and 60°C for 1 minute, every sample was in triplicate (ViiA 7 Real Time PCR System, Applied Biosystems, Foster City, CA,USA). VEGF mRNA level was calculated according to the 2^−(ΔΔCt)^ method and then normalized to β-actin expression.

### Immunohistochemistry

Tumor tissues were first fixed in 10% neutral buffered formalin. After paraffin was removed using xylene series, the slides were rehydrated with ethanol series and incubated with 3% H_2_O_2_ in order to eliminate the endogenous peroxidase activity. After antigen retrieval, slides were incubated with primary antibody CD31 (1:50, Abcam), CD34 (1:100, Abcam), caspase-3 (1:500, Cell Signaling) and Ki67 (1:500, Abcam) at 4°C overnight. The sections were washed with PBS 3 times and incubated with horseradish peroxidase-conjugated secondary antibody (1:100, Cell Signaling) for 30 minutes at room temperature. Signals were visualized by diaminobenzidine reaction and counterstained with hematoxylin. Number of CD31, CD34 stained vessels and caspase-3, Ki67 positive cells were analyzed from 3 random high power fields of each slide. Sections with primary antibody absent and the same concentration of secondary antibody served as negative control.

### Statistical analysis

Experiment data were expressed as mean ± SD. SPSS 17.0 and Sigma Plot 10.0 were used to statistical analysis and plot. Comparisons between treated groups and vehicle control were performed using Independent-Samples T Test. P-value < 0.05 was considered to be significantly different.

## Results

### Combination of quercetin and 2-ME increased inhibition of prostate cancer xenograft tumor growth

**Fig 1:**
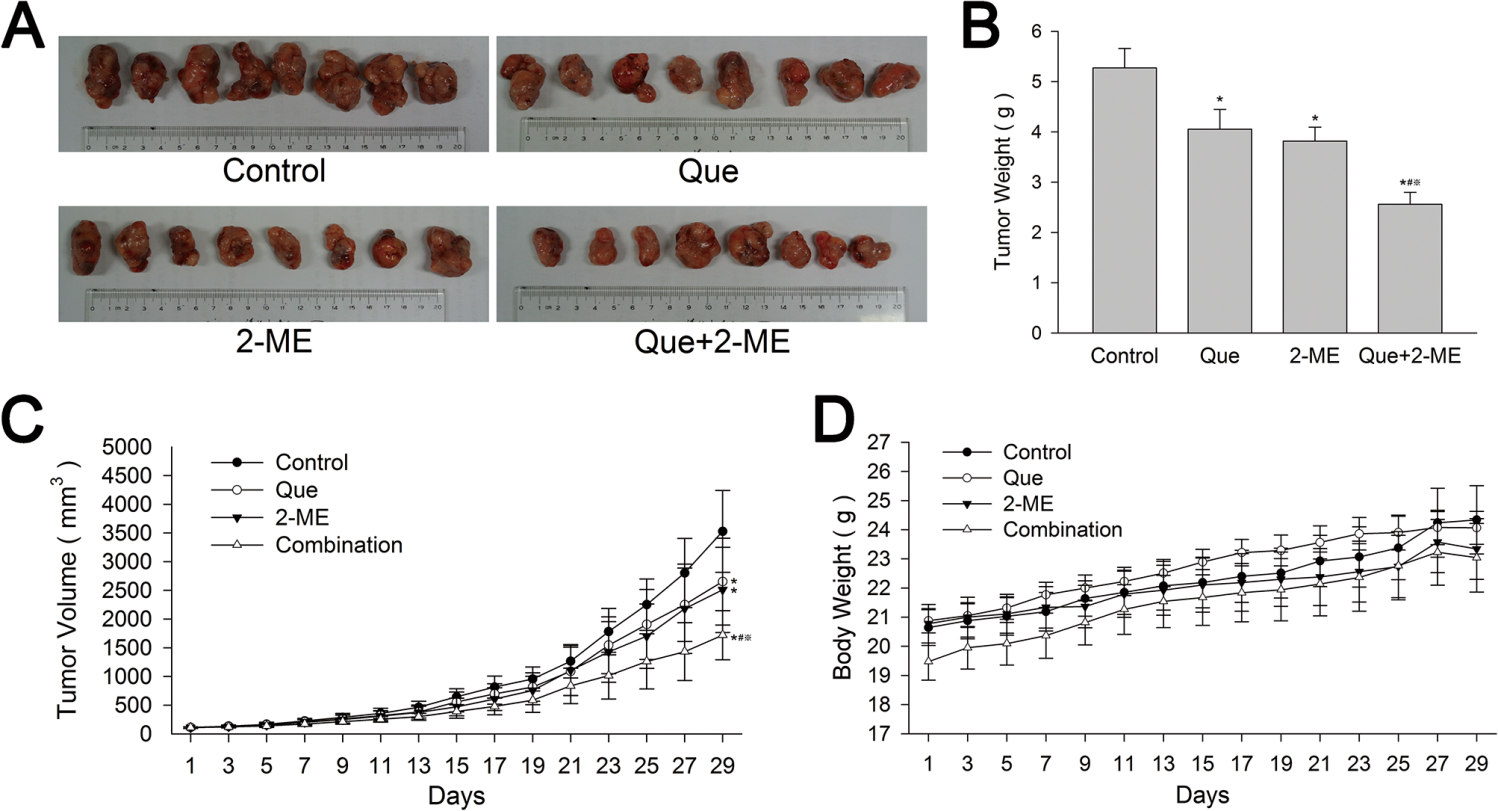
Quercetin combined with 2-ME enhanced inhibition of PC-3 xenograft tumor growth. (A) PC-3 xenograft tumors were smaller in Que or 2-ME treatment group than vehicle control, and the inhibition was more remarkable in Que and 2-ME co-treatment group. Tumor weight (B) and tumor volume (C) were reduced significantly by combination therapy, more obvious than Que or 2-ME alone. There was no significant difference in mouse weight between groups (D). Data are represented as means ± SD. *P<0.05 compared with control, #P<0.05 denotes a significant difference between combination treatment group and quercetin treated group, ※P<0.05 denotes a significant difference between combination treatment group and 2-ME treated group.

**Fig 2:**
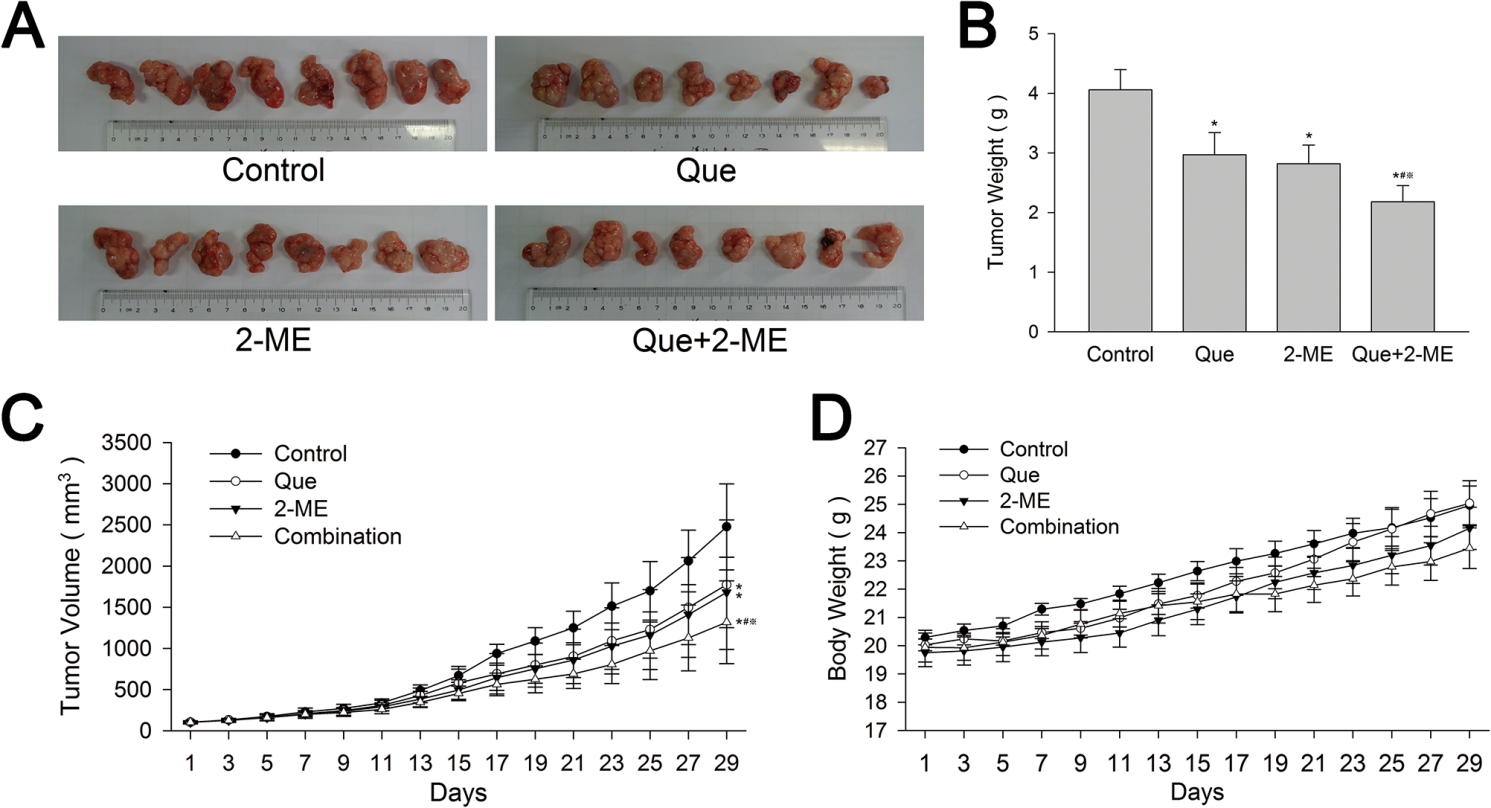
Quercetin combined with 2-ME enhanced inhibition of LNCaP xenograft tumor growth. (A) LNCaP xenograft tumors were smaller in Que or 2-ME treatment group than vehicle control, and the inhibition was more notable in combination treatment group. Que and 2-ME combination therapy reduced tumor weight (B) and tumor volume (C) greatly, more effective than Que or 2-ME alone. No significant influence on mouse weight was observed between groups (D). Data are represented as means ± SD. *P<0.05 compared with control, #P<0.05 denotes a significant difference between combination treatment group and quercetin treated group, ※P<0.05 denotes a significant difference between combination treatment group and 2-ME treated group.

Male BALB/c nude mice with PC-3 and LNCaP cells xenograft tumors were treated with vehicle, Que or 2-ME and their combination and the procedure lasted for 4 weeks. When tumors were taken out, it was obvious that both PC-3 and LNCaP xenograft tumors in Que or 2-ME treatment group were smaller than control, and the inhibition was more remarkable in Que and 2-ME co-treatment group (Figs 1A and 2A). PC-3 xenograft tumors in Que, 2-ME and combination therapy groups were inhibited by 24.8%, 28.9% and 51.3% respectively compared with vehicle control (Fig 1B and 1C), and for LNCaP, the inhibition rate were 28.4%, 32.1% and 46.8% respectively (Fig 2B and 2C). During the whole intervention process, Que, 2-ME and their combination were well tolerated by mice at the selected dose, weight loss in the three treatment groups were not significantly different from control (Figs 1D and 2D). There was no difference in daily food and water consumption between groups as well. Other toxic reaction related with drugs and vehicles such as poor metal state, hematuria were not observed. Significant differences were not found in serum biochemical parameters such as ALT, AST, creatinine and urea nitrogen that reflect drug toxicity on liver and kidney between before and after treatment.

### Effect of quercetin with 2-ME combination treatment on Bax and Bcl-2 protein expression

**Fig 3:**
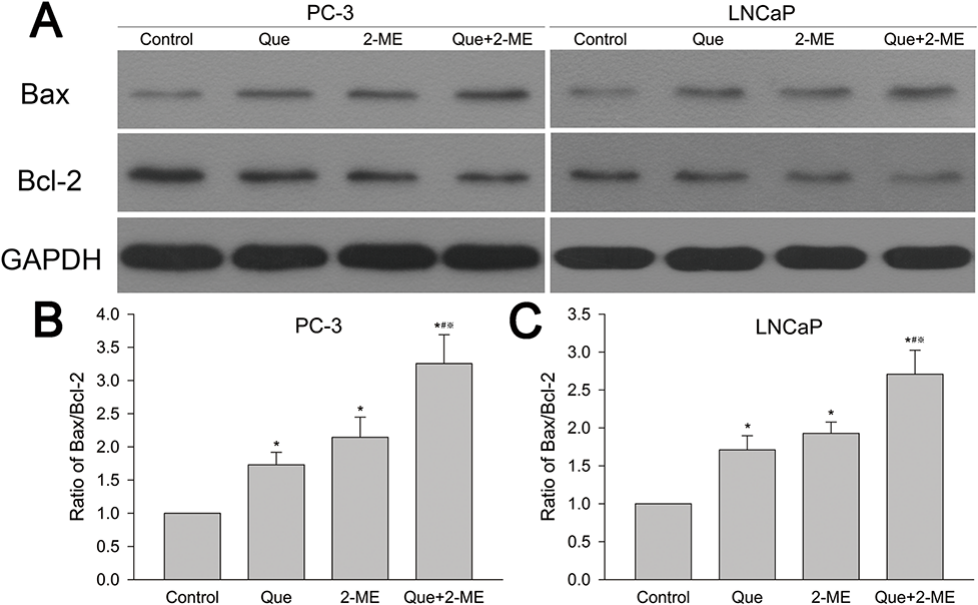
Quercetin combined with 2-ME increased Bax/Bcl-2 ratio in PC-3 and LNCaP xenograft tumor tissues. (A) Western blot detected Bax and Bcl-2 protein expression. (B, C) Bax/Bcl-2 ratio was represented as means ± SD (mean in triplicate). *P<0.05 compared to untreated control, #P<0.05 denotes a significant difference between combination treatment group and quercetin treated group, ※P<0.05 denotes a significant difference between combination treatment group and 2-ME treated group.

Western blot showed that combination of Que and 2-ME enhanced induction of apoptosis by regulating proapoptotic protein Bax and anti-apoptotic protein Bcl-2 expression. As Fig 3A shows, Bax increased and Bcl-2 decreased in single or combined drug treated groups of both PC-3 and LNCaP xenograft tumor tissues. Effect of induced apoptosis was determined by ratio of Bax/Bcl-2 which was increased in Que or 2-ME treated group as compared to control, and combined therapy led to a further increase (Fig 3B and 3C).

### Effect of quercetin with 2-ME combination treatment on the ratio of cleaved caspase-3/caspase-3

**Fig 4:**
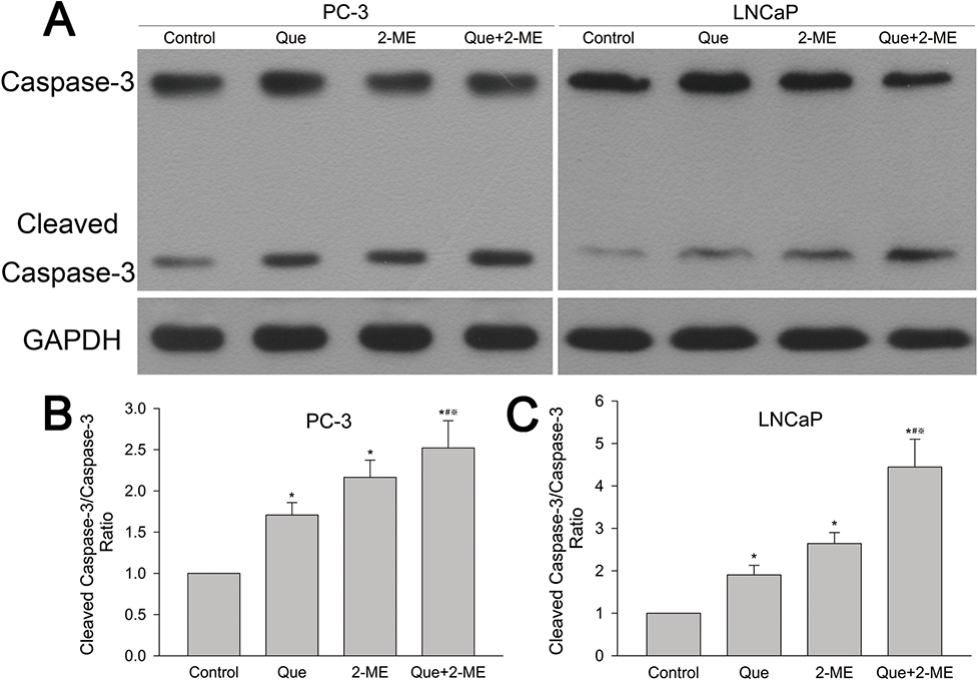
Quercetin combined with 2-ME increased cleaved caspase-3/caspase-3 ratio in PC-3 and LNCaP xenograft tumor tissues. (A) Western blot detected cleaved caspase-3 and caspase-3 protein expression. (B, C) Cleaved caspase-3/caspase-3 ratio was represented as means ± SD (mean in triplicate). *P<0.05 compared to untreated control, #P<0.05 denotes a significant difference between combination treatment group and quercetin treated group, ※P<0.05 denotes a significant difference between combination treatment group and 2-ME treated group.

Effect of quercetin and 2-ME on cleaved caspase-3/caspase-3 ratio was determined by western blot. As shown in Fig 4A and 4B, cleaved caspase-3/caspase-3 ratio elevated in quercetin or 2-ME treated PC-3 tumor tissue and this effect was more obvious in co-treatment group. The same phenomenon was observed in LNCaP tumor tissue (Fig 4A and 4C).

### Effect of quercetin with 2-ME combination treatment on tumor proliferation (Ki67) and apoptosis (caspase-3)

**Fig 5:**
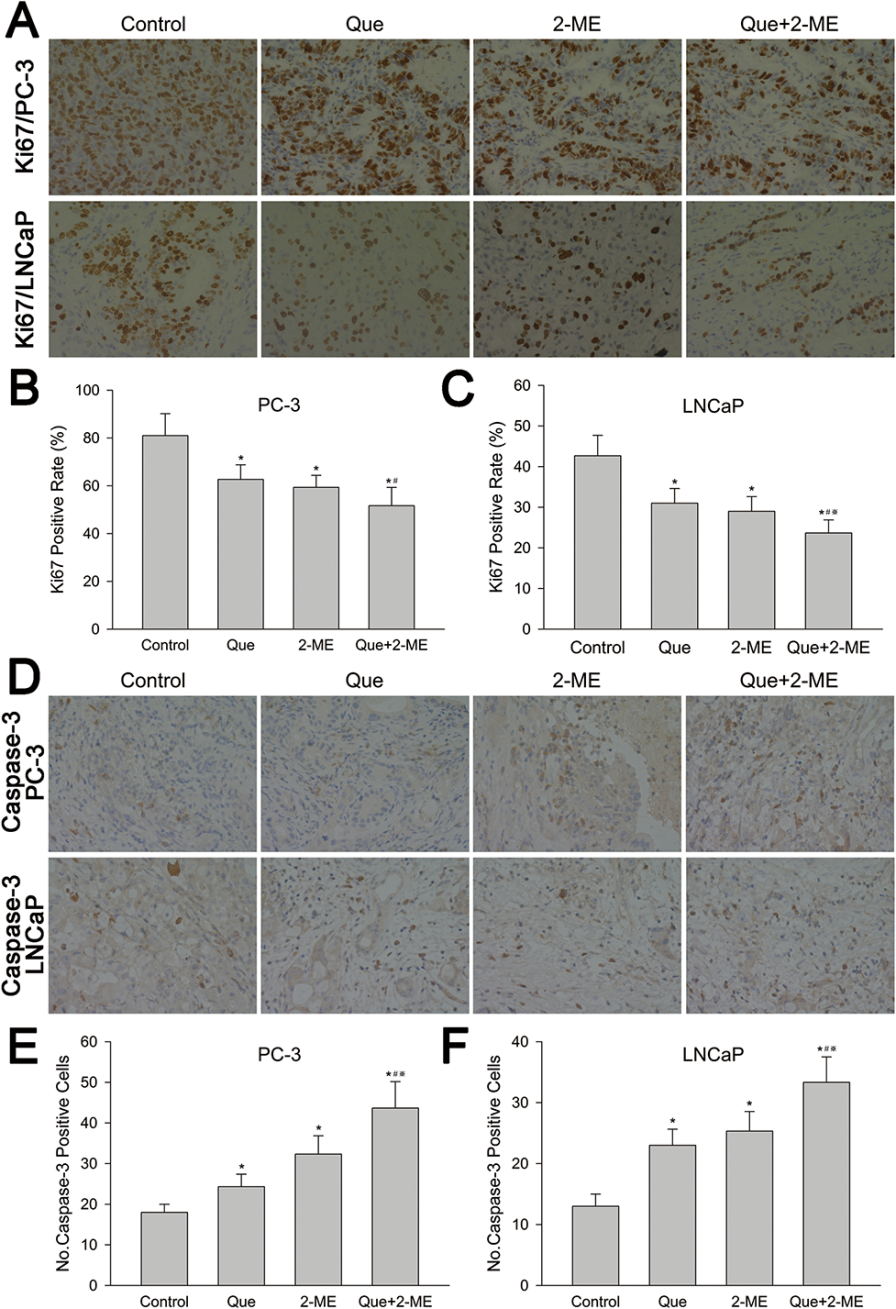
Quercetin combined with 2-ME inhibited proliferation and promoted apoptosis in PC-3 and LNCaP xenograft tumor tissues. (A, D) Immunohistochemical detection showed Ki67 and caspase-3 positive cells in both PC-3 and LNCaP xenograft tumor. Number of Ki67 (B, C) and caspase-3(E, F) positive cells were represented as means ± SD, number was from three random high powered fields per slide (light microscopy, hpf, 400×). *P<0.05 compared to control, #P<0.05 denotes a significant difference between combination treatment group and quercetin treated group, ※P<0.05 denotes a significant difference between combination treatment group and 2-ME treated group.

Immunohistochemical analysis showed that proliferation rate of PC-3 and LNCaP tumor tissue were reduced by 36.2% and 44.5% respectively in co-treatment groups compared with control (p<0.05 and 0.01 respectively), more obvious than quercetin(22.6% for PC-3, 27.3% for LNCaP) and 2-ME (26.7% for PC-3, 32% for LNCaP) alone (Fig 5A–5C). While quercetin or 2-ME single treatment largely increased caspase-3 positive cells (Quercetin: 1.35 fold for PC-3, 1.77 fold for LNCaP. 2-ME: 1.8 fold for PC-3, 1.95 fold for LNCaP, compared with control), co-treatment resulted in more caspase-3 positive cells (2.43 fold for PC-3, 2.56 fold for LNCaP, compared with control) (p<0.01) (Fig 5D–5F). These results indicated that quercetin combined with 2-ME inhibited proliferation and promoted apoptosis of both PC-3 and LNCaP tumor tissue to a larger degree.

### Effect of quercetin with 2-ME combination treatment on phosphatidylinositol 3-kinase (PI3K)/AKT signal pathway

**Fig 6:**
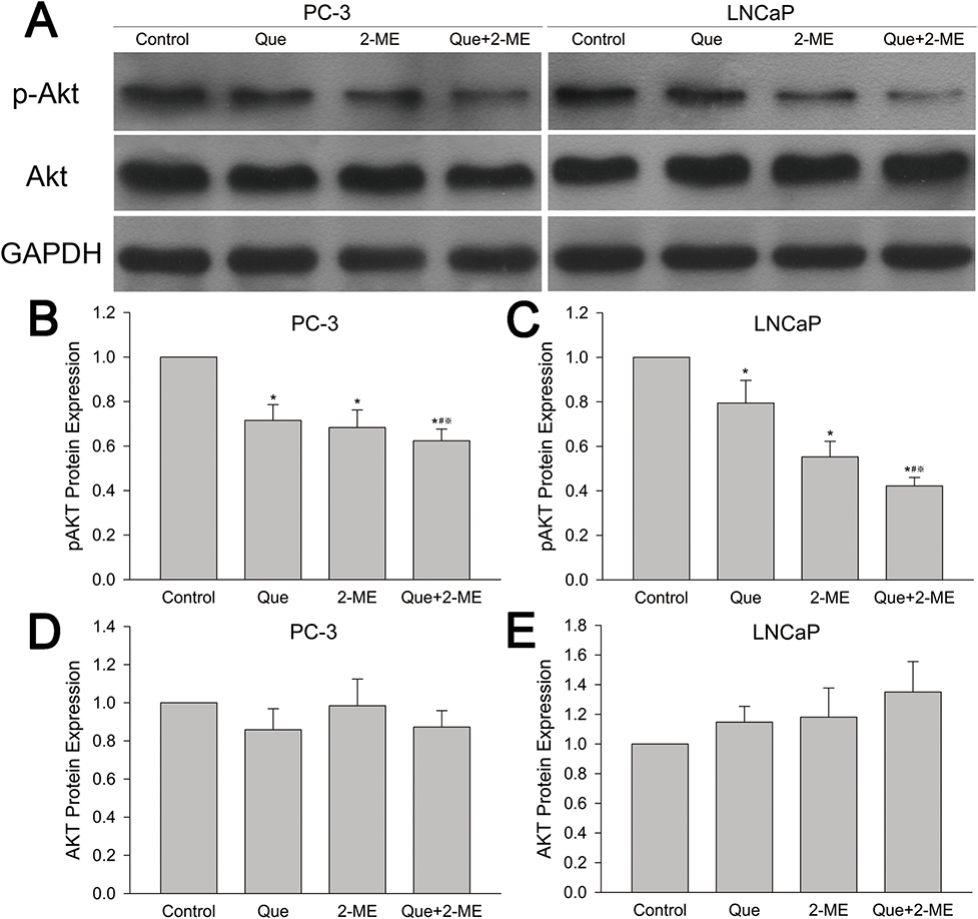
Quercetin combined with 2-ME decreased pAKT protein expression in PC-3 and LNCaP xenograft tumor tissues. (A) pAKT and AKT protein expression were examined by western blot. Values of pAKT (B, C) and AKT (D, E) were represented as means ± SD (values from three independent experiments). *P<0.05 compared to untreated control, #P<0.05 denotes a significant difference between combination treatment group and quercetin treated group, ※P<0.05 denotes a significant difference between combination treatment group and 2-ME treated group.

Western blot revealed that there were no significant difference in AKT protein expression between groups in both PC-3 and LNCaP xenograft tumor tissue (Fig 6A, 6D and 6E). Whereas, pAKT protein was greatly declined in monotherapy groups as compared to vehicle treated group and the inhibition was much more remarkable in quercetin and 2-ME combination treated group (Fig 6A–6C).

### Effect of quercetin and 2-ME combined treatment on VEGF protein and mRNA expression

**Fig 7:**
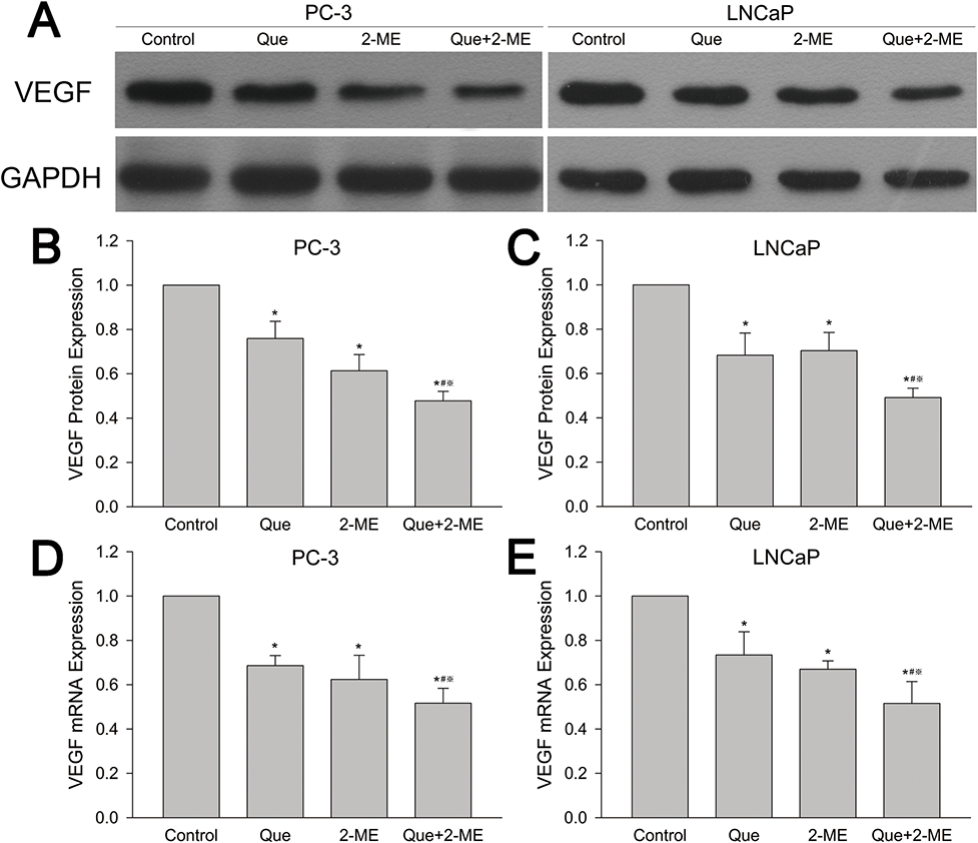
Quercetin combined with 2-ME decreased VEGF protein and mRNA in PC-3 and LNCaP xenograft tumor tissues. VEGF protein (A) and mRNA (D, E) expression were examined by western blot and quantitative real time PCR. Values of VEGF protein (B, C) and mRNA (D, E) were represented as means ± SD (values from three independent experiments). *P<0.05 compared to untreated control, #P<0.05 denotes a significant difference between combination treatment group and quercetin treated group, ※P<0.05 denotes a significant difference between combination treatment group and 2-ME treated group.

From Fig 7A–7C, it could be seen that combination of quercetin and 2-ME significantly reduced VEGF protein expression compared to individual treatment, which in turn exhibited stronger inhibition than vehicle control. As for VEGF mRNA, Q-PCR showed a lower level in individual therapy groups than control, and the suppression effect was further reinforced in co-treatment group whatever in PC-3 or LNCaP tumor tissues (p< 0.01) (Fig 7D and 7E).

### Effect of quercetin with 2-ME combination treatment on microvessel density

**Fig 8:**
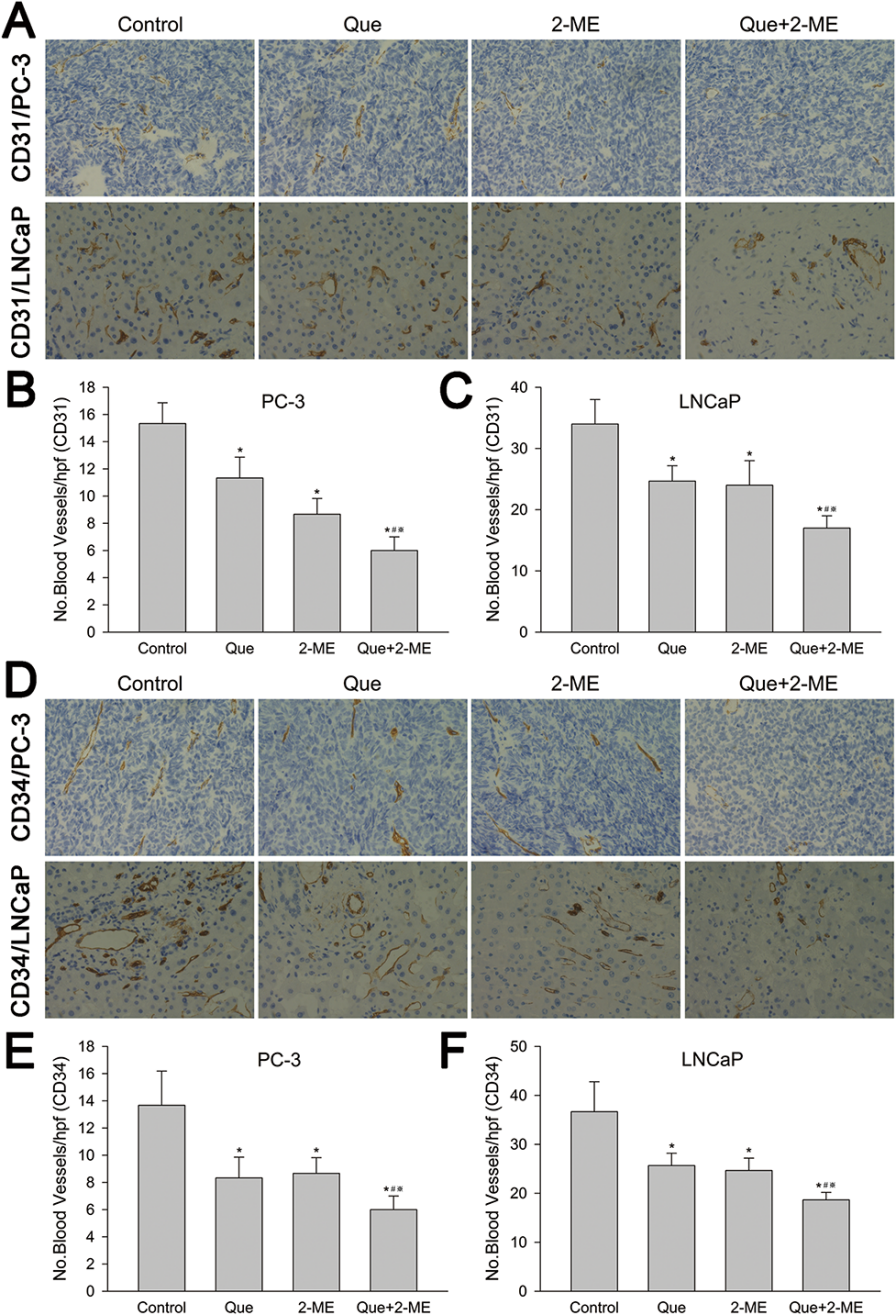
Quercetin combined with 2-ME decreased microvessel density in PC-3 and LNCaP xenograft tumor tissues. (A, D) Immunohistochemical examination exhibited CD31 and CD34 positive vessels in both PC-3 and LNCaP xenograft tumor tissue. Number of CD31 (B, C) and CD34 (E, F) positive vessels were represented as means ± SD, number was from three random high powered fields per slide (light microscopy, hpf, 400×). *P<0.05 compared to control, #P<0.05 denotes a significant difference between combination treatment group and quercetin treated group, ※P<0.05 denotes a significant difference between combination treatment group and 2-ME treated group.

Inhibition effect on microvessel density represented by CD31 and CD34 in PC-3 and LNCaP xenograft tumor tissue of quercetin and 2-ME was further investigated by immunohistochemistry. In PC-3 tumor tissues, combined treatment resulted in a remarkable decrease of CD31 and CD34 (60.87% and 56.1% respectively) expression in comparision with vehicle treated group (P<0.01), while the reduce rate were 26.09% for CD31, 39.02% for CD34 in quercetin applied group and 43.38% for CD31, 36.59% for CD34 in 2-ME used group (Fig 8A, 8B, 8D and 8E). In LNCaP tumor tissues, CD31 and CD34 were declined by 50% and 49.09% respectively in combination treatment group compared with vehicle control (P<0.01), and at the same time, quercetin or 2-ME alone also brought about downregulation of CD31 and CD34 (Quercetin: 27.45% for CD31, 30% for CD34. 2-ME: 29.41% for CD31, 32.73% for CD34) (Fig 8A, 8C, 8D and 8F).

## Discussion and Conclusions

With the increased incidence and mortality of prostate cancer and based on the current adverse treatment situation for castration-resistant prostate cancer (CRPC) [1,4], drug combination has attracted great attention due to the advantage of increased anti-cancer effect, less drug dose, reduced side effects, etc. Herein, following our previous in vitro research [14], we conducted a study of combining quercetin with 2-ME to act on human prostate cancer LNCaP and PC-3 xenograft tumor in male BALB/c nude mouse and found that combined use enhanced inhibition of xenograft tumor growth which was attributed to induction of apoptosis, inhibition of phosphatidylinositol 3-kinase (PI3K)/Akt signal pathway and angiogenesis.

Quercetin and 2-ME are promising anti-prostate cancer substances. As a single agent, quercetin has been verified to inhibit prostate cancer growth both in vitro and in vivo [5,6]. Our previous study also found that quercetin inhibited LNCaP cells growth through down regulation of androgen receptor and its inducible genes [24]. What is more, quercetin is of very low toxicity and rarely produce any side effects even at high dose of 200 mg/kg given to rats and SCID mice [19,20], and clinical trails showed that total consumption of 1000mg quercetin per day could be well tolerated in human not associated with any side effects [25,26]. 2-ME also inhibited growth of prostate cancer cells and xenograft tumor not producing hematological toxicities as other MTAs [10,11,13]. High dose of 150 mg/kg for animals in preclinical trails[13] and 1000 mg or 1500 mg orally four times daily for human in phase I and II trials could be well tolerated without any obvious toxic effects [27,28].

However, despite these inspiring results for prostate cancer, quercetin and 2-ME both have the disadvantage of low bioavailability which discounts their anti-prostate cancer effect. Therefore, they have been combined with other agents, for example, quercetin with green tea, dietary phytoestrogens and tamoxifen [6,20,29,30], 2-ME with albendazole, docetaxel and eugenol [10,13,31], and all showed increased anti-prostate cancer effect even at low dose, which greatly mad up the insufficient bioavailability. Chang et al reported that combination of quercetin and 2-ME increased cytotoxic effect on hepatocellular carcinoma (HCC) cells as compared with quercetin or 2-ME alone [32]. It is speculated that simultaneous use of quercetin and 2-ME would create greater anti-prostate cancer effect with more safety and less toxicity. Our previous in vitro experiment showed that combined use of quercetin and 2-ME exhibited synergistic inhibition of proliferation and induction of apoptosis in both androgen-dependent LNCaP and androgen-independent PC-3 human prostate cancer cell lines as compared to single drug, and it was attributed to arresting cell cycle and decreasing Bcl-2/Bax ratio [14]. But it is unknown about the in vivo effect on prostate cancer and in vitro results can not be directly applied to clinical. Hence, we combined quercetin with 2-ME to act on LNCaP and PC3 xenograft tumor and found that combination treatment inhibited xenograft tumor growth by 46.8% for LNCaP and 51.3% for PC3 as compared to vehicle control, more effective than quercetin (28.4% for LNCaP, 24.8% for PC3) or 2-ME (32.1% for LNCaP, 28.9% for PC3) alone. Moreover, they were well tolerated and no obvious toxic reaction was observed in the whole treatment process. It is verified that combination of quercetin and 2-ME could inhibit prostate cancer growth in vivo in a larger degree with more effectiveness and safety.

Targeted therapy has been considered critical to chemotherapeutic effect of natural products and phytochemicals [33]. Quercetin and 2-ME can act on some signal pathways leading to effectively inhibiting prostate cancer growth especially when they are combined.

Bcl-2 family proteins, including antiapoptotic Bcl-2 and proapoptotic Bax, are important mediator of mitochondrial apoptosis pathway. Activated Bax helps second mitochondria derived activator of caspase (Smac) and cytochrome-c release that thereby activate caspase-3 into cleaved caspase-3 leading to cell apoptosis, while antiapoptotic protein Bcl-2 prevents Bax activation by binding and sequestering it letting cancer cells escape from apoptosis [30,34]. Thus, Bax/Bcl-2 ratio and the level of cleaved caspase-3 indicate the therapeutic response and are very important in apoptosis induction [33]. Quercetin increased Bax/Bcl-2 ratio by 1.3-fold of androgen-sensitive LAPC-4 prostate cancer cell xenograft tumor [6]. Kumar et al reported that quercetin therapy significantly increased Bax/Bcl-2 ratio and caspase-3 activity in PC-3 cells [30]. 2-ME inhibited proliferation and induced apoptosis of LNCaP and PC3 cells in vitro and in vivo through phosphorylation of Bcl-2 [35,36]. Our present study demonstrated that quercetin and 2-ME used alone increased proapoptotic protein Bax, decreased antiapoptotic protein Bcl-2 and elevated Bax/Bcl-2 ratio thereby leading to caspase-3 activation, which in turn induced apoptosis and inhibited xenograft tumor growth. These results were further confirmed by immunohistochemical analysis of caspase-3 and Ki67 positive cells. Moreover, all these effects were enhanced by combination of quercetin and 2-ME resulting in tumor inhibition to a larger extent.

AKT, a serine/threonine protein kinase belonging to PI3K/AKT survival pathway, plays an important role in cell survival and apoptosis when it is activated into phosphorylated AKT (pAKT). pAKT is upregulated and plays an important role in survival and proliferation of human prostate cancer cells [37]. High level of pAKT is related with high-grade and high Gleason score of prostate tumors and serves as an independent predictor of biochemical recurrence [38]. Therefore, AKT has been considered as a promising therapeutic target for prostate cancer. Kim et al showed that quercetin reduced pAkt protein facilitating TRAIL- induced apoptosis in LNCaP and DU145 cell lines [39]. Lee et al revealed that quercetin reduced pAKT level resulting in suppression of phosphorylated Bad and influencing relationship between Bcl-xL and Bax, which then increased activity of Bax and finally led to LNCaP cell apoptosis [40]. 2-ME sensitized PC-3 cells to fas mediated apoptosis via inhibition of pAKT [41]. In our experiment, quercetin and 2-ME can respectively decreased pAKT protein expression in both LNCaP and PC-3 xenograft tumor tissue, but pAKT decreased much more obviously when the combination treatment was applied. Through inhibition of pAKT signal pathway, tumor growth was suppressed more effectively.

Angiogenesis refers to generation of new blood vessels from preexisting vascular system ensuring tumors to obtain enough oxygen and nutrients and is regulated by many angiogenic factors, among which vascular endothelial growth factor (VEGF) is the most crucial one [42]. Anti-cancer drugs can inhibit angiogenesis by downregulating VEGF through which tumor growth is inhibited. Pratheeshkumar et al found that quercetin reduced VEGF levels in PC-3 cells and inhibited blood vessel formation both in vitro and in vivo. In this way, PC-3 cells and xenograft tumors were inhibited [43]. Quercetin also significantly reduced VEGF secretion in LNCaP cells [44]. Mabjeesh et al illuminated that 2-ME remarkably decreased VEGF level in a dose dependent way in PC-3 cells, and then angiogenesis and tumor growth were inhibited [45]. In addition, measuring microvessel density (MVD) using endothelial markers has been widely applied for evaluation of angiogenesis in many previous studies [46]. Immunohistochemistry and antibodies against the endothelial cell markers CD31 and CD34 have frequently been used in many articles to investigate the pathological significance of MVD in prostate cancer [47,48,49]. Our research demonstrated that quercetin and 2-ME combination decreased both VEGF protein and mRNA dramatically in PC-3 and LNCaP xenograft tumors, which was more obvious compared to quercetin or 2-ME alone. Furthermore, immunohistochemical results showed that microvascular density represented by CD31 and CD34 was less in single drug treated groups than vehicle control, and it was further reduced in co-treatment group. All these verified that combination of quercetin and 2-ME could suppress both PC-3 and LNCaP xenograft tumor growth more remarkably by inhibiting angiogenesis via reducing VEGF protein and mRNA expression.

In summary, our research here demonstrated that combination treatment of quercetin and 2-ME enhanced inhibition of both androgen-dependent LNCaP and androgen- independent PC-3 human prostate cancer cells xenograft tumor growth in vivo and it was related with enhanced induction of apoptosis, increased inhibition of phosphatidylinositol 3-kinase (PI3K)/Akt signal pathway and angiogenesis. These in vivo results can provide a solid foundation for the future studies on this novel therapeutic regimen and in some degree raise the possibility of applying combined use of quercetin and 2-ME to prevent and treat prostate cancer in human.
